# Can Endothelin-1 Levels in Patients with Esophageal Variceal Bleeding at Admission Predict Rebleeding Within 5 Days?

**DOI:** 10.5152/tjg.2024.23028

**Published:** 2024-02-01

**Authors:** Shaker Wagih Shaltout, A. El Messery, Ahmed Elshabrawi, Ahmed I. Amin, Mostafa H. Elshennawy, Metwaly Ibrahim Mortada, Walid ElSherbiny, Hatem Elalfy, Dina Elhammady

**Affiliations:** 1 Department of Tropical Medicine, Port Said University Faculty of Medicine, Port Said, Egypt; 2 Department of Endemic Medicine, Mansoura University Faculty of Medicine, Mansoura, Egypt; 3 Department of Internal Medicine, Port Said University Faculty of Medicine, Port Said, Egypt; 4 Department of Tropical Medicine, Mansoura University Faculty of Medicine, Mansoura, Egypt; 5 Division of Hematology, Department of Clinical Pathology,Mansoura University Faculty of Medicine, Egypt

**Keywords:** Cirrhosis, gastrointestinal bleeding, varices, rebleeding, endothelin-1

## Abstract

**Background/Aims:**

Portal hypertension complicating liver cirrhosis is associated with vascular resistance, possibly due to overexpression of humoral vasoconstrictors, including endothelin. The study aimed to evaluate the efficacy of serum endothelin-1 levels as a noninvasive predictor of early esophageal rebleeding (within 5 days) following endoscopic treatment.

**Materials and Methods:**

Of the patients presented to the endoscopy unit at Mansoura University Hospital, 50 patients were chosen for this study on the basis of endoscopically proven acute esophageal variceal bleeding consequent to hepatitis C viral infection complicated by liver cirrhosis and portal hypertension. Routine laboratory parameters and serum endothelin-1 levels were assessed prior to endoscopic treatment. Patients were divided into 2 groups depending on the development of early postendoscopic rebleeding. Group A consisted of 16 patients who developed rebleeding, while group B included 34 patients who did not. Statistical analysis was performed to determine the predictors of rebleeding.

**Results:**

Multivariate logistic regression demonstrated that endothelin-1 level (*P* < .001) and serum albumin level (*P* = .04) were independent risk factors for early rebleeding. The most efficient cutoff value for endothelin-1 levels in predicting variceal rebleeding within the 5 days after endoscopic intervention was 65.29, which had an 88.2% specificity, 87.5% sensitivity, 88% accuracy, and area under the curve value of 0.89. In addition, hemoglobin, albumin, and creatinine levels were significantly different between bleeding and nonrebleeding groups (*P* = .03, *P* = .014, and *P* <.001, respectively), as was the duration of hospital stay (*P* < .001).

**Conclusion:**

Serum endothelin-1 levels appear to be a reliable, practical, noninvasive predictor of early variceal rebleeding and related comorbidities such as the severity of kidney affection and duration of hospital stay.

**Main Points**

- Rebleeding is a frequent problem after endoscopic treatment of bleeding esophageal varices.
- Endothelin-1 has been demonstrated to participate in the regulation of portal vein pressure and the motion of sinusoidal endothelial fenestrae after binding to its receptors.
- Our findings demonstrated that endothelin-1 levels were a reliable, practical, noninvasive predictor of early variceal rebleeding and related comorbidities such as the severity of renal impairment and duration of hospital stay.

## Introduction

Esophageal or gastric variceal bleeding as a complication of portal hypertension accounts for the rise in cirrhotic patient hospital admission, liver transplantation, and death.^1^ Variceal rebleeding is a frequent problem following endoscopic treatment of esophageal varices, occurring at an incidence of 30% within 6 weeks of the initial bleed and increasing to 60% within 1 year, with a mortality rate of 34% within 12 months.^2^

To date, the most accurate predictor of initial variceal bleeding is via indirect measurement of portal hypertension through assessment of the hepatic venous pressure gradient (HVPG), where an HVPG above 12 mm Hg is indicative that a bleed will most likely occur.^3^ Other predictors of potentially impending variceal hemorrhage are the presence of Child–Pugh B or C cirrhosis, denoting functionally compromised and decompensated liver function, respectively, as well as the presence of red wale marks on endoscopically observed varices.^4^

Although it is widely accepted that endoscopic variceal ligation (EVL) is an effective maneuver for the prevention of variceal bleeding,^5^ the occurrence of early recurrent bleeding post-EVL is a severe complication with fatal consequences. This rebleeding occurs from 24 hours to 14 days following endoscopy, most likely due to the presence of an unhealed residual ulcer left behind by the spontaneous slippage of the ligation bands. Currently, there is limited research available on the predictive role of potential factors in the occurrence of variceal rebleeding following endoscopic intervention, including the presence of a previous attack of upper variceal bleeding and local factors such as peptic esophagitis or a large number of varices. Similarly, the presence of a high platelet ratio index score or altered coagulation tests have also been suggested as indicators of future variceal rebleeding.^6^ Therefore, the seriousness of this side effect necessitates further research.

Portal hypertension complicating liver cirrhosis is associated with vascular resistance possibly attributed to the overexpression of humoral vasoconstrictors such as norepinephrine, endothelin (ET), and angiotensin-II.^7^ Endothelin-1 (ET-1) binds to both ET-A receptors found on vascular smooth muscle cells, where they mediate vasoconstriction, and ET-B receptors on endothelial cells, where they lead to NO release through stimulation of endothelial NO synthase (eNOS). Accordingly, the role of ET-1 in liver disease may be profound, especially in disorders characterized by circulatory dysfunction such as portal hypertension or ischemia.^8^

Since the principal site of synthesis and action of endothelin-1 (ET-1) is the liver, its increased plasma levels and overexpression in cases of cirrhosis demonstrate the marked contribution of ET-1 to the pathogenesis of portal hypertension in these patients.^9^ Endothelin-1 has been demonstrated to participate in the regulation of portal vein pressure and the rearrangement of sinusoidal endothelial fenestrae after binding to its receptors.^10^ Based on these findings, the aim of this study was to evaluate the level of serum endothelin-1 as a predictor of both early variceal rebleeding following endoscopic treatment and development of comorbidities.

## MATERIALS and Methods

This prospective cohort study was performed on patients presenting with an acute attack of hematemesis to the endoscopy unit at Mansoura University Hospital between March 2019 and November 2019. Ethical approval was obtained from the Institutional Review Board at Mansoura University (Code Number: R.21.04.1310). Written informed consent was obtained from all patients.

### Target Subjects and Groups

Out of 217 patients with upper gastrointestinal tract (GIT) bleeding admitted to the Endemic Medicine Department, 50 patients with first endoscopically proven acute esophageal variceal bleeding due to liver cirrhosis and portal hypertension complicating hepatitis C viral infection were enrolled in this prospective study. All included patients were admitted within 24 hours of the bleeding attack. Patients who had a previous history of variceal bleeding were excluded.

Patients were divided into groups A and B depending on whether or not they experienced an attack of rebleeding within 5 days following endoscopic treatment. Rebleeding was identified by the occurrence of recurrent melena, hematemesis, or bloody fluid drained through a nasogastric tube between 24 hours and 5 days after the endoscopy; a decrease in hemoglobin of at least 2 g/dL; requirement for a transfusion of more than 2 units of concentrated red blood cells within 24 hours; or the occurrence of hypovolemic shock. Group A was the rebleeding group and included 16 patients, while the 34 patients who did not develop recurrent bleeding were assigned to group B.

### Endoscopic, Biochemical, and Sonographic Evaluation

Routine laboratory investigations and serum ET-1 levels were assessed on admission. Supportive measures were performed to control the bleeding. The endoscopic assessment was carried out within 12 hours after admission while the patients were already receiving the vasoactive drug (octreotide [50-100 μg/h]), prophylactic antibiotic (ceftriaxone 1 g/24 h), and blood transfusion for those with hemoglobin level below 8 g/dL.

### Statistical Analysis

Collected data were prepared, tabulated, and statistically analyzed using Statistical Package for Social Science Version 16.0 (SPSS Inc.; Chicago, IL, USA). Number (percent) was used to present categorical data, and mean (SD) or median (interquartile range) was used to present continuous data following the results of Shapiro–Wilk test for the assumption of normal distribution of data. Significance testing was done using chi-square test, while Fisher’s exact test was used for categorical data, Welch’s *t*-test for parametric data, and Mann–Whitney *U*-test for nonparametric variables. Furthermore, Spearman correlation was used to test for association between nonparametric data.

To determine predictors of rebleeding among studied cases, multivariate analysis was performed. Variables found to have a significant association in univariate analysis were entered in the regression model. Receiver operator curve (ROC) analysis was used to determine the validity of endothelin-1 in identifying cases prone to rebleeding. The level of significance was set at *P* < .05%.

## Results

This study was conducted on 50 cirrhotic patients, with ages ranging from 44 to 77 years with a mean of 55.4 ± 7.92. Twenty-eight (56%) of the patients were male, and 22 (44%) were female. According to the Child–Pugh classification, 18 patients were class A, 22 were class B, and 10 were class C, with a mean score of 7.86 ± 2 for all classes. All patients were admitted to Mansoura University Hospital with acute upper gastrointestinal (GI) bleeding and had a hospital stay ranging from 2 to 20 days (mean 7.22 ± 4.96). Thirty-four patients (68%) received a blood transfusion, and 26 patients (52%) required intensive care unit (ICU) admission (Table 1).

Upper GI endoscopy showed esophageal varices in all of the patients. Nineteen patients (38%) had F2 grade esophageal varices, and 31 (62%) had F3 grade. Thirty-nine patients (78%) had high-risk stigmata for bleeding at endoscopy.

During the hospital stay, recurrence of bleeding developed in only 16 patients (32%), characterized by the mean age of 60 ± 7.6 years, with 7 patients (43.8%) being male. It was determined that the rebleeding cases were all caused by esophageal variceal bleeding through endoscopy or clinical manifestations. On the other hand, those who did not develop rebleeding had a mean age of 53.2 ± 7.21, and 21 patients were male (61.8%). Hemoglobin, albumin, and creatinine levels were significantly different between the 2 groups (*P* = .03, *P* = .014, and *P* <.001, respectively). There was no significant difference between the Child–Pugh scores and the number of variceal columns of the 2 groups (*P* = .277 and *P* = .073, respectively). Patients with early rebleeding had a higher mean grade of esophageal varices (F3) than those nonbleeding cases (F2) (*P* = .054). A highly significant difference between groups was found regarding the duration of hospital stay (*P* < .001) characterized by a mean of 11.19 ± 3.82 days for those who developed recurrent bleeding and 5.35 ± 4.33 days for those who had no rebleeding (Table 1).

The median endothelin-1 (ET-1) level was 25.22 for the group that had no rebleeding and 90.08 for the rebleeding group, demonstrating a significant difference between the 2 groups (*P* = .01).

**Figure 1.**
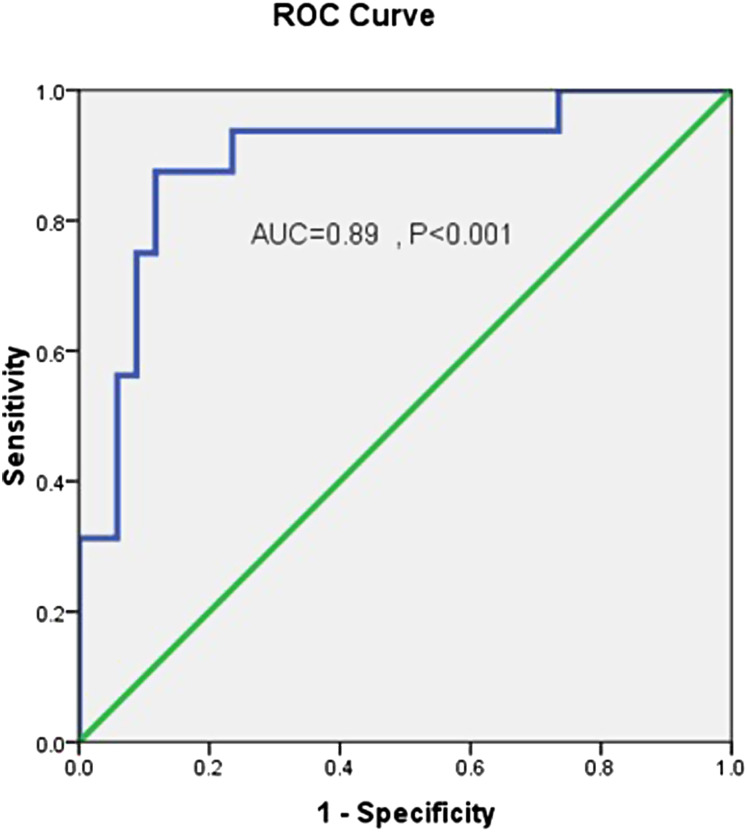
ROC curve of endothelin for differentiating rebleeding among studied cases. ROC, receiver operator curve.

ET-1 level demonstrated an odds ratio of 1.05, with a 95% CI of 1.02-1.08, and *P* < .001 for differentiating rebleeding among studied cases. ET-1, at a cutoff value of 65.29, had an 88.2% specificity, 87.5% sensitivity, 88% accuracy, and area under the curve (AUC) of 0.89 for predicting variceal rebleeding within 5 days after endoscopy (Table 2). The ROC curve is demonstrated in Figure 1.

**Figure 2.**
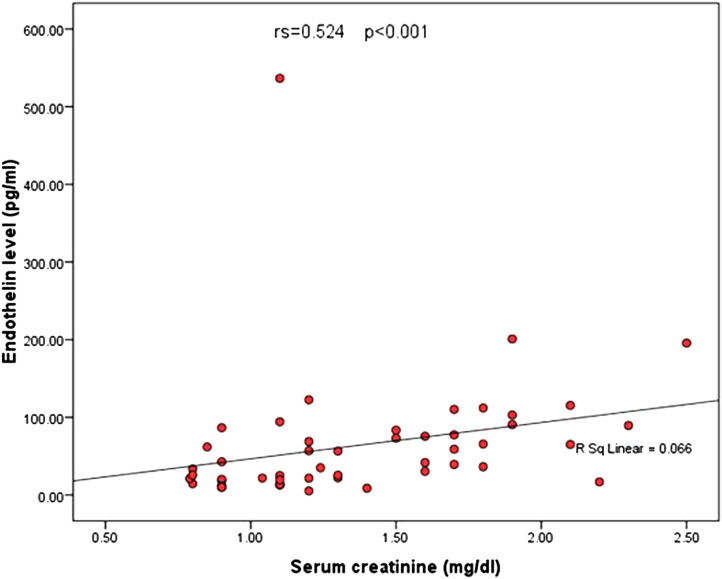
Correlation between ET-1 level and the degree of renal impairment. ET-1, endothelin-1.

**Figure 3.**
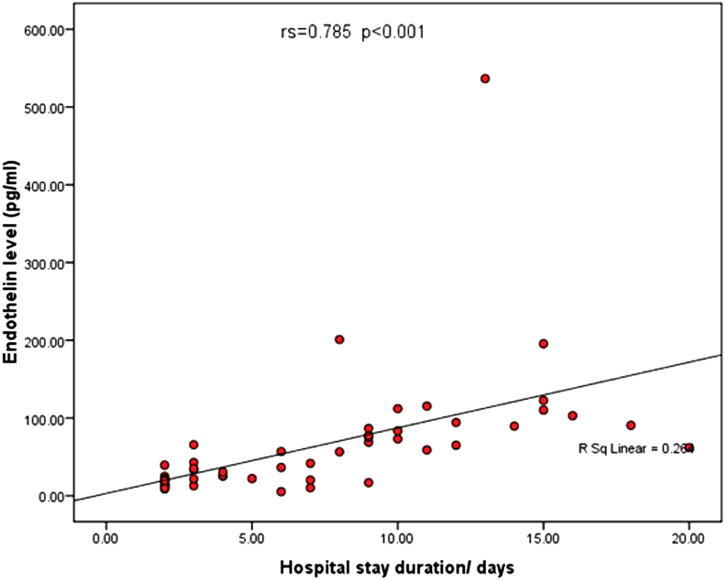
Correlation between ET-1 level and the duration of hospital stay. ET-1, endothelin-1.

Using stepwise multivariate logistic regression analysis, levels of ET-1 were found to be significantly correlated with the severity of portal hypertensive endoscopic findings, especially with regards to variceal cord number (*P* = .006) and complications following rebleeding, such as the drop in hemoglobin level (*P* = .002) and degree of renal impairment (*P* < .001) (Tables 3 and 4). Notably, ET-1 level was significantly correlated with the duration of hospital stay (*P* < .001) (Tables 5-7, Figures 2 and 3). All included patients were discharged home after improvement.

## Discussion

Although variceal bleeding of either primary or secondary origin is effectively prevented by EVL, postendoscopy early variceal bleeding is a life-threatening complication occurring at a rate from 7.6% to 19% that requires further research.^6,11^ Early reports on initial rebleeding following EVL showed that potential risk factors included ascitic volume, number of rubber bands used in ligation, variceal severity, and Prothrombin Time (PT) prolongation.^11^

The endothelin (ET) family consists of 3 members designated as ET-1, ET-2, and ET-3. As a potent vasoconstrictor, endothelin-1 (ET-1) is broadly expressed in numerous tissues, including blood vessels, as well as several organs, such as the heart, lungs, and liver. Levels of ET-1 have been shown to be closely related to the extent of liver fibrosis and, hence, the accompanying portal hypertension.^12^ Acting as a paracrine hormone, plasma levels of ET-1 possibly depict an overabundance of peptides produced locally.^13^

The present study showed that the level of endothelin-1 (ET-1) was a predictor for early variceal rebleeding occurring within 5 days of endoscopic intervention most effectively at the cutoff value of 65.29. In addition, the level of ET-1 was found to be significantly higher in patients showing endoscopic findings suggestive of more advanced portal hypertension, including variceal cord number and complications following rebleeding, such as decreased hemoglobin level and extent of renal impairment. Furthermore, a significant correlation was also found between levels of ET-1 and the duration of patient hospital stay.

Correspondingly, a correlation between decreased portal vein ET-1 level and lowered portal vein pressure gradient was demonstrated by Meng et al^14^ following TIPS insertion. Similar findings were also affirmed in another study by Kawanaka et al^15^ showing that splenectomy resulted in decreased hepatic concentrations of ET-1 associated with a reduction in portal venous pressure in cases of portal hypertension complicating liver cirrhosis. Because an enlarged spleen may overexpress ET-1 by vascular endothelial cells, splenectomy may eliminate spleen-derived ET-1, thereby decreasing systemic and splanchnic circulation.

On an experimental scale, the increase in portal venous pressure observed in laboratory rats may be due to the overexpression of ET-1 in cirrhotic liver tissue as detected by immunohistochemical analysis, especially in sinusoidal endothelial cells inside the regenerating nodules, in addition to the increase in ET-1 plasma level in these experimental subjects.^10^

In addition to ET-1 level, the current study also showed that serum albumin level, as a single parameter of the Child–Pugh scoring system, was found to be independent risk factor for prediction of early variceal rebleeding following intervention with EVL. Although the Child–Pugh score showed no relation to rebleeding in this study, several previous studies have reported the Child–Pugh score to be an independent risk factor for death from rebleeding. The Child–Pugh scoring system for liver function was shown to be an independent risk factor of rebleeding following endoscopy in the study by Yang et al,^16^ with Berreta et al^17^ also showing that patients classed as Child–Pugh C were more at risk of death from rebleeding.

Our study’s small sample size of cases of rebleeding was one of its limitations. In the future, more cases should be gathered from multiple centers. Additionally, not all cases of rebleeding were diagnosed by endoscopy, which prevented us from conducting a more comprehensive analysis.

Serum endothelin-1 level appears to be a reliable, practical, noninvasive predictor of early variceal rebleeding and related comorbidities such as the severity of kidney affection and duration of hospital stay.

## Figures and Tables

**Table 1.** Patient Characteristics

	n	%
Age	55.42 ± 7.92	44-70
Sex		
Male	28	56%
Female	22	44%
Cirrhosis	50	100%
Splenomegaly	50	100%
Ascites	25	50%
PVT	6	12%
Varices grade		
F2	19	38%
F3	31	62%
PHG	38	76%
Variceal risky signs	39	78%
Bleeding recurrence		
No	34	68%
Yes	16	32%
Blood transfusion		
No	16	32%
Yes	34	68%
ICU admission	26	52%

ICU, intensive care unit; PVT, portal vein thrombosis; PHG, portal hypertensive gastropathy.

**Table 2.** Validity of Endothelin in Differentiating Rebleeding Cases

	AUC (95% CI)	*P*	Cutoff Points	Sensitivity	Specificity	PPV	NPV	Accuracy
Endothelin	0.89 (0.79-0.99)	<.001^*^	≥65.29	87.5%	88.2%	77.8%	93.8%	88.0%

AUC, area under the curve; NPV, negative predictive value; PPV, positive predictive value. *Statistically significant.

**Table 3.** Univariate Analysis for Detection of Risk Factors of Rebleeding

	Nonrebleeding, n = 34 (68.0%)	Rebleeding, n = 16 (32.0%)	Test of Significance	Odds Ratio (95% CI)
Endothelin				
Median	25.22	90.08	*z* = 4.45	1.05 (1.02-1.08)
(Range)	(5.28-112.02)	(16.85-536.6)	*P* < .001^*^	
(IQR)	(14.36-46.31)	(70.55-120.78)		
Number of varices	3.0 (1.0-4.0)	3.0 (2.0-4.0)	*z* = 1.79, *P* = .07	1.98 (0.96-4.12)
1	4 (11.8)	0 (0.0)	MC, *P* = .21	
2	10 (29.4)	2 (12.5)		
3	19 (29.4)	7 (43.8)		
4	10 (29.4)	7 (43.8)		
Grade of varices				
F2 (R)	16 (47.1)	3 (18.8)	χ^2^ = 3.70	1
F3	18 (52.9)	13 (81.2)	*P* = .054	3.85 (0.93-16.01)
Risky signs				
Absent (R)	10 (29.4)	1 (6.2)	χ^2^ = 3.4	1
Present	24 (70.6)	15 (93.8)	*P* = .06	6.25 (0.73-53.89)
Portal vein thrombosis				
Absent (R)	30 (88.2)	14 (87.5)	FET	1
Present	4 (11.8)	2 (12.5)	*P* = 1.0	1.07 (0.18-6.56)
Child score				
A (R)	13 (38.2)	5 (31.2)	χ^2^ = 1.86	1
B	16 (47.1)	6 (37.5)	*P* = .39	0.97 (0.24-3.93)
C	5 (14.7)	5 (31.2)		2.6 (0.52-13.04)
Platelet count				
Median	69.0	64.5		
*z* = 0.74, *P* = .46	0.99 (0.97-1.02)			
		(Range)			(11.0-129.0)	(45.0-132.0)
		(IQR)	(53.75-85.75)	(53.5-76.5)
Bilirubin (mg/dL)				
Median	1.15	1.65			*z* = 2.23, *P* = .02^*^	1.82 (0.87-3.81)
(Range)	(0.3-4.2)	(0.4-4.3)				
(IQR)	(0.9-1.73)	(1.53-2.18)				
Albumin (g/dL), mean ± SD	2.92 ± 0.31	2.67 ± 0.35	*t* = 2.55, *P* = .01^*^	0.10 (0.01-0.71)

^*^ Statistically significant.

***χ*** **^2^**, chi-square test; FET, Fisher’s exact test; IQR, interquartile range; MC, Monte Carlo test; R, reference; *t*, Student’s *t*-test; *z*, Mann–Whitney *U*-test.

**Table 4.** Multivariate Analysis for Detection of Predictors of Rebleeding

Predictors	*β*	*P*	Adjusted Odds Ratio
Endothelin	0.05	.001^*^	1.05 (1.02-1.08)
Bilirubin (mg/dL)	1.14	.14	1.32 (0.07-1.77)
Albumin (g/dL)	−5.17	.04^*^	0.006 (0.001-0.83)

Overall percent predicted = 86.0%; Model *χ* ^2^ = 31.45

^*^ *P* < 0.001.

**Table 5.** Correlation of Endothelin Levels with a Degree of Renal Impairment (Creatinine), Duration of Hospital Stay, and Hemoglobin Level

Predictors		Endothelin Level
Hospital stay (duration in days)	*R*_s_	0.785
	*P*	<.001
Serum creatinine (mmol/L)	*R*_s_	0.524
	*P*	<.001
Hemoglobin (HB) level	*R*_s_	−0.424
	*P*	.002

*R*_s_, Spearman correlation coefficient.

**Table 6.** Linear Regression for Predicting Endothelin Level (After Log Transformation)

Predictors	*β*	*t*	*P*
Hospital stay (duration in days)	0.052	5.45	<.001^*^
Serum creatinine (mmol/L)	0.165	1.57	<.001^*^

*Statistically significant. **Prediction equation**

Endothelin level = 1.007 + 0.052 × hospital stay (days) + 0.165 × serum creatinine (mg/dL).

*R* ^2^ = 0.753.

**Table 7.** Correlation of Endothelin Level with Endoscopic and Laboratory Findings

	Endothelin Level
Age	
	* R*_s_	0.492
*P*	.000
Hospital stay (duration in days)	
	*R*_s_	0.785
*P*	.000
Number of variceal columns	
	*R*_s_	0.384
*P*	.006
Creatinine	
	*R*_s_	0.524
*P*	.000
Alanine transaminase	
	*R*_s_	0.201
*P*	.161
Child score	
	*R*_s_	0.203
*P*	.157
Total leukocyte count	
	*R*_s_	−0.143
*P*	.323
Hemoglobin	
	*R*_s_	−0.424
*P*	.002
Platelets	
	*R*_s_	−0.235
*P*	.101
Albumin	
	*R*_s_	−0.239
*P*	.095
Bilirubin	
	*R*_s_	0.229
*P*	.110
